# Elevated hematologic ratios are correlated with acne severity: a national, retrospective cohort study

**DOI:** 10.3389/fmed.2024.1475117

**Published:** 2024-10-31

**Authors:** Vered Wiesel, Sarah Weissmann, Bracha Cohen, Inbal Golan-Tripto, Amir Horev

**Affiliations:** ^1^Faculty of Health Sciences, Ben Gurion University of the Negev, Beer Sheva, Israel; ^2^Clinical Research Unit, Soroka University Medical Center, Beer Sheva, Israel; ^3^Pediatric Pulmonary Unit, Soroka University Medical Center, Beer Sheva, Israel; ^4^Pediatric Dermatology Service, Soroka University Medical Center, Beer Sheva, Israel

**Keywords:** acne vulgaris, hematologic ratios, NLR, PLR, acne, ELR, ENR

## Abstract

**Background:**

Prior studies demonstrated conflicting results regarding hematologic ratios in acne patients. We sought to further characterize hematologic ratios in acne patients, according to demographics and acne severity.

**Methods:**

National, retrospective cohort study of 122,822 patients using medical records from 2005 to 2024 of patients insured with the largest public healthcare organization in Israel, Clalit Health Maintenance Organization.

**Results:**

Moderate–severe acne patients had higher neutrophil-to-lymphocyte ratio (NLR) and platelet-to-lymphocyte ratio (PLR) than mild acne patients at diagnosis and 12–18 months before diagnosis. A multivariable regression confirmed the significance of the correlation of increased NLR and PLR with acne severity. Adults and females had higher NLR and PLR than children and males, respectively, at diagnosis, and 12–18 months before diagnosis.

**Conclusion:**

Acne severity was significantly associated with elevated NLR and PLR. NLR and PLR may also serve as indicators of upcoming acne severity, as they were elevated 12–18 months before diagnosis. These biomarkers may contribute to the diagnosis, management, and follow-up of patients with acne.

## 1 Introduction

Acne vulgaris is one of the most common skin conditions (1). It affects a staggering 9.4% of people of all ages (1, 2), with 20% of patients suffering from moderate to severe acne (3). Up to 85% of individuals aged 12–24 years suffer from acne (1). The incidence of acne peaks at the age of 15 years and declines throughout late adolescence (4). However, prevalence continues into the second and third decades of life in 64% and 43% of individuals, respectively (1). There is a debate in the literature about whether acne disproportionally affects females or males (4–7), though severe acne may disproportionally affect males (8).

Since the pathophysiology of acne involves activation of the immune system, specifically, neutrophils and helper T cells (9–11), hematologic ratios, specifically neutrophil-to-lymphocyte ratio (NLR) and platelet-to-lymphocyte ratio (PLR), may be effective as accessible biomarkers of acne’s inflammation. These ratios have been proposed as useful in the assessment of severity and prognosis of other dermatologic conditions, including psoriasis (12), urticaria (13, 14) atopic dermatitis (15), and vitiligo (16), as well as non-dermatologic conditions (17–20). Notably, they were shown to be higher 12–18 months before the diagnosis of disease in some dermatologic conditions (12, 16). With regards to acne and hematologic ratios, there are very few conflicting studies in the literature, with cohorts ranging from 20 patients to 65 patients (21–24).

To our knowledge, this is the first study to characterize hematologic markers for acne in a large, national cohort that includes patients younger than 16 years old. These ratios may be important considerations in assessing acne severity and prognosis, and thus management.

## 2 Materials and methods

### 2.1 Study design

This retrospective, national cohort study included patients of all ages with acne between 2005 and 2024. We extracted data from MDClone, a data-sharing platform with data-synthesizing capabilities, regarding the medical records of patients insured in the Clalit Health Maintenance Organization (HMO). The Clalit HMO is the largest public healthcare organization in Israel, serving around 4,700,000 people, which is about half of the population of Israel, across 27 hospitals and more than 1,500 community-based clinics in Israel. The study was approved by the local Ethics Committee of Soroka University Medical Center (No. 0434-15-SOR).

### 2.2 Study population and blood count parameters

We included all patients insured by Clalit HMO who were diagnosed with acne according to ICD-10 codes. We compared patients according to age, gender, socioeconomic status, ethnicity, complete blood counts and hematologic ratios. The socioeconomic score was determined by the National Institute for Statistics according to the zip code of the patient. We calculated hematologic ratios from complete blood counts by dividing the relevant variables. For example, NLR was calculated by dividing neutrophils by lymphocytes.

We analyzed patients’ hematologic ratios within 30 days of diagnosis with acne, and 12–18 months before diagnosis. We compared children (ages 0–18 years) vs. adults, females vs. males, and patients with mild vs. moderate–severe acne. We defined patients as having moderate–severe acne if they fulfilled one of the following conditions: (1) If they took systemic medication for acne, including isotretinoin, antibiotics, spironolactone, or oral contraceptive pills. (2) If they were diagnosed with acne fulminans or acne conglobate. (3) If they visited the emergency department for their acne.

Lastly, we excluded patients if they had no blood work done within 30 days of diagnosis with acne or 12–18 months before diagnosis. We excluded patients if they had a history of acute or chronic infections, malignancies, or surgery within the past 30 days of their blood test. We excluded patients if they were diagnosed with a systemic disease that leads to acne, such as polycystic ovary syndrome, Cushing syndrome, Behçet’s disease, androgen secreting tumors, PAPA syndrome, Alpert syndrome, and acromegaly.

### 2.3 Statistical analysis

We analyzed continuous variables using the Student’s *t*-test or Mann–Whitney U test, and categorical variables using the Chi-squared test or Fisher’s exact test. We performed a multivariable logistic regression adjusted for age, gender, socioeconomic status, and ethnicity for each hematological ratio independently. We considered *p*-values < 0.05 statistically significant. All statistical analyses were completed using R software (version 4.0.2).

## 3 Results

### 3.1 Children vs. adults

The study included 70,427 adults and 52,395 children aged 0–18 years with acne (Table 1). The proportion of females was significantly higher in the adults compared to the children (mean: 77.5% vs. 58.4%, *p* < 0.001). Adults with acne had a mean age at diagnosis of 29.83 years, while children were diagnosed at a mean age of 15.2 years (*p* < 0.001). Notably, adults with acne were predominantly from a higher socioeconomic status than children, and of Jewish ethnicity (*p* < 0.001 for both). Importantly, adult patients exhibited a significantly higher prevalence of moderate–severe acne (mean: 6.6% of adults compared to 4.7% of children, *p* < 0.001).

**Table 1:** Clinical, demographic, and laboratory characteristics of adults and children with acne.

	Adults	Children	*p*-value
*N*	70,427	52,395	
**Gender (%)**			<0.001
Female	54,563 (77.5)	30,591 (58.4)	
Male	15,862 (22.5)	21,803 (41.6)	
**Age at diagnosis [mean (SD)]**	29.83 (13.16)	15.20 (2.16)	<0.001
**Socioeconomic score (%)**			<0.001
High	12,854 (18.3)	8,706 (16.6)	
Medium	34,866 (49.5)	26,018 (49.7)	
Low	16,419 (23.3)	13,861 (26.5)	
**Ethnicity (%)**			<0.001
Arab	21,307 (30.3)	17,381 (33.2)	
Jewish	44,904 (63.8)	30,852 (58.9)	
Other	2,960 (4.2)	2,150 (4.1)	
No data	1,256 (1.8)	2,012 (3.8)	
**Severe acne (%)**	4,638 (6.6)	2,485 (4.7)	<0.001
Neutrophils [mean (SD)]	4.08 (1.67)	3.74 (1.65)	<0.001
Platelets [mean (SD)]	258.14 (66.2)	272.50 (66.04)	<0.001
Eosinophils [mean (SD)]	0.20 (0.15)	0.23 (0.19)	<0.001
Lymphocytes [mean (SD)]	2.29 (0.74)	2.54 (0.82)	<0.001
Monocytes [mean (SD)]	0.45 (0.17)	0.47 (0.202)	<0.001
NLR [mean (SD)]	1.90 (1.11)	1.60 (1.10)	<0.001
PLR [mean (SD)]	127.10 (45.25)	114.83 (41.08)	<0.001
ELR [mean (SD)]	0.09 (0.07)	0.09 (0.07)	<0.001
ENR [mean (SD)]	0.05 (0.05)	0.07 (0.07)	<0.001
EMR [mean (SD)]	0.47 (0.42)	0.51 (0.44)	<0.001
NLR 12–18 months before [mean (SD)]	2.14 (1.66)	1.74 (1.75)	<0.001
PLR 12–18 months before [mean (SD)]	127.10 (54.92)	122.10 (53.59)	<0.001
ELR 12–18 months before [mean (SD)]	0.09 (0.07)	0.10 (0.08)	<0.001
ENR 12–18 months before [mean (SD)]	0.05 (0.05)	0.07 (0.07)	<0.001
EMR 12–18 months before [mean (SD)]	0.47 (0.41)	0.54 (0.49)	<0.001

SD, Standard Deviation; NLR, Neutrophil Lymphocyte Ratio; PLR, Platelet Lymphocyte Ratio; ELR, Eosinophil Lymphocyte Ratio; ENR, Eosinophil Neutrophil Ratio; EMR, Eosinophil Monocyte Ratio.

Compared to children, adults had higher NLR (mean: 1.90 vs. 1.60, *p* < 0.001) and PLR (mean: 121.70 vs. 114.83, *p* < 0.001). Adults had higher levels of neutrophils, and lower levels of lymphocytes and platelets, compared to children. Furthermore, adults had higher NLR (mean: 2.14 vs. 1.74, *p* < 0.001) and PLR (mean: 127.10 vs. 122.10, *p* < 0.001) compared to the children 12–18 months before diagnosis. Eosinophils, monocytes, eosinophils to lymphocyte ratio (ELR), eosinophil-to-neutrophil ratio (ENR), and eosinophil-to-monocyte ratio (EMR) showed statistically significant results of limited clinical significance.

### 3.2 Females vs. males

This analysis included 85,154 females with acne and 37,665 males with acne (Table 2). Females were older at diagnosis than males (mean: 24.28 years vs. 22.02 years, *p* < 0.001), and the proportion of children was lower among females (mean: 35.9% vs. 57.9%, *p* < 0.001). Socioeconomic score and ethnicity were both significantly different between the females and males, as females tended to be from a lower socioeconomic status, and of Arab ethnicity (*p* < 0.001 for both). The severity of acne did not differ between males and females (*p* = 0.541).

**Table 2:** Clinical, demographic, and laboratory characteristics of females and males with acne.

	Female	Male	*p*-value
*N*	85,154	37,665	
**Age at diagnosis [mean (SD)]**	24.28 (12.04)	22.02 (13.4)	<0.001
**Socioeconomic score (%)**			<0.001
High	14,661 (17.2)	6,898 (18.3)	
Medium	41,448 (48.7)	19,435 (51.6)	
Low	21,735 (25.5)	8,545 (22.7)	
**Ethnicity (%)**			<0.001
Arab	28,078 (33.0)	10,610 (28.2)	
Jewish	51,413 (60.4)	24,340 (64.6)	
Other	3,486 (4.1)	1,624 (4.3)	
No data	2,177 (2.6)	1,091 (2.9)	
**Severe acne (%)**	4,915 (5.8)	2,208 (5.9)	0.541
**Children (%)**	30,591 (35.9)	21,803 (57.9)	<0.001
Neutrophils [mean (SD)]	3.98 (1.65)	3.90 (1.72)	<0.001
Platelets [mean (SD)]	269.24 (66.54)	248.28 (63.53)	<0.001
Eosinophils [mean (SD)]	0.20 (0.16)	0.24 (0.19)	<0.001
Lymphocytes [mean (SD)]	2.35 (0.76)	2.47 (0.84)	<0.001
Monocytes [mean (SD)]	0.44 (0.17)	0.51 (0.20)	<0.001
NLR [mean (SD)]	1.82 (1.10)	1.68 (1.15)	<0.001
PLR [mean (SD)]	123.77 (44.41)	107.45 (39.62)	<0.001
ELR [mean (SD)]	0.08 (0.07)	0.10 (0.08)	<0.001
ENR [mean (SD)]	0.05 (0.05)	0.07 (0.07)	<0.001
EMR [mean (SD)]	0.48 (0.42)	0.51 (0.46)	<0.001
NLR 12–18 months before [mean (SD)]	2.03 (1.64)	1.98 (1.87)	0.072
PLR 12–18 months before [mean (SD)]	128.19 (54.24)	117.36 (54.80)	<0.001
ELR 12–18 months before [mean (SD)]	0.09 (0.07)	0.11 (0.09)	<0.001
ENR 12–18 months before [mean (SD)]	0.05 (0.05)	00.07 (0.07)	<0.001
EMR 12–18 months before [mean (SD)]	0.48 (0.42)	0.54 (0.47)	<0.001

SD, Standard Deviation; NLR, Neutrophil Lymphocyte Ratio; PLR, Platelet Lymphocyte Ratio; ELR, Eosinophil Lymphocyte Ratio; ENR, Eosinophil Neutrophil Ratio; EMR, Eosinophil Monocyte Ratio.

Females with acne had higher NLR (mean: 1.82 vs. 1.68, *p* < 0.001) and PLR (mean: 123.77 vs. 107.45, *p* < 0.001) compared to males with acne. Females with acne had higher levels of neutrophils and platelets, and lower levels of lymphocytes than males with acne. Moreover, 12–18 months before diagnosis, PLR was higher in females than males (mean: 128.19 vs. 117.36, *p* < 0.001), but NLR was not significantly different between the genders (*p* = 0.072).

### 3.3 Mild vs. moderate–severe acne patients

Stratification by severity revealed significant differences in demographics (Table 3). This analysis included 115,699 patients with mild acne and 7,123 patients with moderate–severe acne. Socioeconomic status and ethnicity were significantly different between the moderate–severe and mild acne groups, as moderate–severe acne patients had a higher proportion of people from high socioeconomic class and Jewish ethnicity (*p* < 0.001 and *p* = 0.024, respectively). The proportion of children was lower among the moderate–severe acne patients (34.9% vs. 43.1%, *p* < 0.001), and patients with moderate–severe acne were diagnosed at an older age compared to patients with mild acne (mean: 28.26 vs. 23.30 years, *p* < 0.001). There was no gender disparity between the patients with mild and moderate–severe acne.

**Table 3:** Clinical, demographic, and laboratory characteristics of patients with mild and moderate–severe acne.

	Mild	Moderate–severe	*p*-value
*N*	115,699	7,123	
Gender (%)			0.75
Female	80,239 (69.4)	4,915 (69.0)	
Male	35,457 (30.6)	2,208 (31.0)	
Age at diagnosis [mean (SD)]	23.30 (11.98)	28.26 (17.28)	<0.001
Socioeconomic score (%)			0.024
High	20,291 (17.5)	1,269 (17.8)	
Medium	57,256 (49.5)	3,628 (50.9)	
Low	28,621 (24.7)	1,659 (23.3)	
Ethnicity (%)			<0.001
Arab	36,482 (31.5)	2,206 (31.0)	
Jewish	71,210 (61.5)	4,546 (63.8)	
Other	4,877 (4.2)	233 (3.3)	
No data	3,130 (2.7)	138 (1.9)	
Children (%)	49,910 (43.1)	2,485 (34.9)	<0.001
Neutrophils [mean (SD)]	3.93 (1.63)	4.30 (2.12)	<0.001
Platelets [mean (SD)]	263.27 (65.84)	265.69 (73.64)	0.056
Eosinophils [mean (SD)]	0.21 (0.17)	0.22 (0.17)	<0.001
Lymphocytes [mean (SD)]	2.38 (0.78)	2.34 (0.85)	0.008
Monocytes [mean (SD)]	0.45 (0.18)	0.47 (0.22)	<0.001
NLR [mean (SD)]	1.76 (1.09)	2.01 (1.52)	<0.001
PLR [mean (SD)]	118.48 (43.11)	123.48 (51.48)	<0.001
ELR [mean (SD)]	0.09 (0.07)	0.10 (0.08)	0.002
ENR [mean (SD)]	0.06 (0.06)	0.06 (0.07)	<0.001
EMR [mean (SD)]	0.49 (0.43)	0.51 (0.49)	<0.001
NLR 12–18 months before [mean (SD)]	2.00 (1.65)	2.19 (2.14)	<0.001
PLR 12–18 months before [mean (SD)]	125.17 (53.60)	130.41 (64.27)	<0.001
ELR 12–18 months before [mean (SD)]	0.09 (0.07)	0.10 (0.09)	<0.001
ENR 12–18 months before [mean (SD)]	0.06 (0.06)	0.06 (0.06)	0.381
EMR 12–18 months before [mean (SD)]	0.49 (0.43)	0.51 (0.48)	0.097

SD, Standard Deviation; NLR, Neutrophil Lymphocyte Ratio; PLR, Platelet Lymphocyte Ratio; ELR, Eosinophil Lymphocyte Ratio; ENR, Eosinophil Neutrophil Ratio; EMR, Eosinophil Monocyte Ratio.

Patients with moderate–severe acne had higher NLR (mean: 2.01 vs. 1.76, *p* < 0.001) and PLR (mean: 123.48 vs. 118.48, *p* < 0.001) than patients with mild acne. Patients with moderate–severe acne had higher levels of neutrophils and lower levels of lymphocytes than patients with mild acne. Platelets were not significantly different between the mild and moderate–severe acne patients. 12–18 months before diagnosis, NLR (mean: 2.19 vs. 2.00, *p* < 0.001) and PLR (mean: 130.41 vs. 125.17, *p* < 0.001), were higher in moderate–severe acne patients than mild acne patients 12–18 months before diagnosis.

### 3.4 Multivariable logistic regression analysis for acne severity

We chose to conduct a multivariable logistic regression analysis for acne severity with NLR and PLR, as they showed the most clinically relevant statistical differences (Table 4). When considering patients with moderate–severe acne, there were statistically significant increases in NLR and PLR, corresponding with increased severity. NLR (odds ratio (OR) 1.11, confidence interval (CI) 1.13) and PLR (OR 1.18, CI 1.24), demonstrated statistically significant associations with acne severity (both *p* < 0.001).

**Table 4:** Severe acne patients’ odds of having higher blood count parameters.

Hematologic ratio	OR^a^	95% CI^a^	*p*-value
NLR	1.11	1.09, 1.13	<0.001
PLR	1.18	1.12, 1.24	<0.001

a OR, Odds Ratio; CI, Confidence Interval.

## 4 Discussion

In this study, we found that elevated NLR and PLR were significantly associated with severity of acne. Previous studies about hematologic ratios and acne severity showed conflicting results, with smaller sample sizes. One study of 76 patients aged 16–35 years showed that NLR was not elevated in patients with severe-very severe acne compared to patients with mild–moderate acne (21). Another study showed no differences in any complete blood count value or hematologic ratio, including NLR and PLR, between 10 severe acne patients, 10 mild acne patients, and 10 healthy controls (22). By contrast, other studies, with cohorts of 65 or fewer patients, showed that NLR and neutrophils were elevated in acne patients compared to healthy controls (23, 24). The elevated NLR and PLR may be related to the presence of Cutibacterium acnes, which has been implicated in the pathogenesis of inflammatory acne (25). Cutibacterium acnes leads to T-cell mitogenic activity (25). These lymphocytes enter the skin lesions of patients with acne, and thus may be decreased in the peripheral blood. Furthermore, Cutibacterium acne is known to increase neutrophil activity and chemotaxis (26). Since acne is an inflammatory disease (10), the slightly elevated platelets may be related to platelets’ role in immunothrombosis and chronic inflammation (26). Overall, our study expanded on previous studies about hematologic ratios and acne and utilized a much larger sample size of more than a hundred thousand patients.

In our study, we noted that moderate–severe patients had an increase in NLR and PLR 12–18 months before acne diagnosis. This contributes to the notion that acne is an inflammatory disease before the development of clinical lesions (27). Inflammatory changes have been seen in uninvolved skin of acne patients before the formation of lesions. For instance, both T lymphocytes and the pro-inflammatory cytokine IL-1 have been shown to infiltrate healthy skin of patients with acne prior to the formation of lesions (10, 28). Since IL1 is associated with leukocyte chemotaxis (29), it is possible that these lymphocytes infiltrate the site of the acne lesion as it forms, and thus decrease relatively in the peripheral blood, corresponding with elevated NLR and PLR. Overall, these hematological changes may imply that PLR and NLR should be considered as indicators for acne prognosis and severity, much before the development of clinical lesions.

In our study, we saw adults with acne had higher NLR and PLR than children with acne. Post adolescence acne is more likely to consist of inflammatory lesions (30). In studies examining biopsies of inflammatory acne lesions, IL-8, a pro-inflammatory cytokine crucial for the chemotaxis of neutrophils toward the acne unit, has been shown to be upregulated, compared to biopsies of uninvolved skin from the same patients (31, 32). Neutrophils, in particular, have been shown to be elevated inside 24-h acne pustules, with an even higher elevation shown in 72-h acne lesions (33). Hence, NLR may be higher in adults due to their pre-disposition for inflammatory, neutrophil-rich acne.

Lastly, in our study, females had higher NLR and PLR, despite no significant differences in the severity of their acne. The literature is inconclusive about whether, in the general population, healthy females and males have naturally different hematologic ratio profiles (34), though it is known that hematologic ratios can vary with age and race (34–36). In particular, NLR has been shown to increase with older age, potentially due to a greater disposition to inflammation and disease as age increases (35, 37). A study of Chinese females and males showed a trend of increasing NLR and PLR up to age 40–59, with a subsequent decrease, in females, while there were little to no age specific differences in males (34). Thus, the differences between the female and male populations of our study may be explained by the higher proportion of adults in the female population of our study.

To the best of our knowledge, this study is the first study to analyze NLR, PLR, ELR, ENR, and EMR in a national cohort of acne patients, and in a cohort that includes children younger than 16 years, nevertheless, we must acknowledge some limitations. First, as this study is a retrospective study, there may be potential biases pertaining to data collection and especially acne severity, since there is still debate in the literature regarding the classification of acne and treatment of acne based on classification (38–40). This is especially relevant in our cohort because we included patients who were diagnosed with acne by primary care physicians and board-certified dermatologists, who may employ different acne diagnosis criteria, leading to a lower prevalence of moderate–severe acne patients within our cohort than expected. As a result, the use of ICD-10 codes in our study, though practical, may not fully reflect the clinical complexity of the condition. To mitigate this limitation, we have used clear criteria for acne severity, as detailed in the methods section. Second, complete blood counts are not part of the routine diagnosis of acne. While lab work may be monitored prior to and during initiation of treatment with isotretinoin, this practice remains controversial (41, 42), and complete blood count analysis is not typically part of the diagnostic workup of mild acne. Therefore, it is possible that the acne patients had an underlying condition warranting a complete blood count analysis. It is known that many conditions may lead to changes in hematologic ratios (12–17). Additionally, in patients with moderate–severe acne, bloodwork may be part of the workup for systemic diseases that involve acne. To minimize this bias, we excluded patients with histories of acute or chronic infections, malignancies, or surgery, and systemic diseases that lead to acne, such as polycystic ovary syndrome and Cushing syndrome. Despite this, it is still possible that we have included patients with other conditions that may affect blood count values. Thirdly, there are other confounding factors, including stress, diet, and use of certain medications, which may affect NLR and PLR, and were not accounted for in this current study. These variables are likely to be present in both the mild and moderate–severe acne patients, though due to the retrospective nature of the study, it is impossible to extrapolate to which degree. Future, prospective, longitudinal studies of hematologic ratios may account for these factors with questionnaires regarding participants’ stress levels and diets. Lastly, while the lab values were taken 30 days before and after the diagnosis of acne according to ICD-10 codes, it could not be determined whether the disease was present at that time.

## 5 Conclusion

In this national retrospective cohort study, we saw significant elevations of NLR and PLR in patients with moderate–severe acne compared to patients with mild acne, both at diagnosis and 12–18 months before diagnosis. According to our multivariable regression model, acne severity was significantly associated with higher NLR and PLR. These biomarkers may contribute to the diagnosis, management, and follow-up of patients with acne. Future studies regarding the mechanism of the changes in these hematologic ratios are warranted, with longitudinal design and additional populations.

## Data Availability

The original contributions presented in the study are included in the article/supplementary material, further inquiries can be directed to the corresponding author.
